# Rapamycin supplementation of *Drosophila melanogaster* larvae results in less viable adults with smaller cells

**DOI:** 10.1098/rsos.230080

**Published:** 2023-06-21

**Authors:** Ewa Szlachcic, Maciej J. Dańko, Marcin Czarnoleski

**Affiliations:** ^1^ Life History Evolution Group, Institute of Environmental Sciences, Faculty of Biology, Jagiellonian University, Kraków, Poland; ^2^ Max Planck Institute for Demographic Research, Rostock, Germany

**Keywords:** ageing, cell size, life history, mortality, survivorship, TOR

## Abstract

The intrinsic sources of mortality relate to the ability to meet the metabolic demands of tissue maintenance and repair, ultimately shaping ageing patterns. Anti-ageing mechanisms compete for resources with other functions, including those involved in maintaining functional plasma membranes. Consequently, organisms with smaller cells and more plasma membranes should devote more resources to membrane maintenance, leading to accelerated intrinsic mortality and ageing. To investigate this unexplored trade-off, we reared *Drosophila melanogaster* larvae on food with or without rapamycin (a TOR pathway inhibitor) to produce small- and large-celled adult flies, respectively, and measured their mortality rates. Males showed higher mortality than females. As expected, small-celled flies (rapamycin) showed higher mortality than their large-celled counterparts (control), but only in early adulthood. Contrary to predictions, the median lifespan was similar between the groups. Rapamycin administered to adults prolongs life; thus, the known direct physiological effects of rapamycin cannot explain our results. Instead, we invoke indirect effects of rapamycin, manifested as reduced cell size, as a driver of increased early mortality. We conclude that cell size differences between organisms and the associated burdens of plasma membrane maintenance costs may be important but overlooked factors influencing mortality patterns in nature.

## 1. Introduction

Longevity-oriented research is strongly motivated by our human perspective [1–4], with societies directing impressive amounts of resources to healthcare systems. For example, globally, the maintenance of healthcare systems required 8 trillion US dollars in 2016, which is predicted to rise to 15 trillion US dollars by 2050 [5]. How well organisms survive, and thus for how long they thrive in the environment to pass their gene copies to subsequent generations, is also a key element shaping Darwinian fitness. Not surprisingly, factors that affect mortality rates in natural populations have attracted continued interest in biological research, especially in the fields of ecology and evolution [4,6]. A complete understanding of mortality patterns in nature requires a simultaneous consideration of different mortality types [6,7], which have either an extrinsic or intrinsic nature [8–10]. In general, intrinsic sources of mortality relate to the ability to cope with the metabolic demands imposed by the molecular repair and maintenance of tissue and organ function and are therefore linked to the accumulation of molecular cellular damage and a reduction in organismal performance with age [6,11–13] (see also [14] for other potential mechanisms of ageing). Consequently, with increasing exposure to internal sources of mortality, e.g. as ageing effects progress through life, organisms also become more vulnerable to external mortality factors, e.g. predation and parasitism, which can directly connect the two sources of mortality in natural populations [7] (see also [15,16] for theoretical consideration of the problem).

Intrinsic forms of mortality, including senescence, occur universally among multicellular and even single-celled organisms (e.g. [13,17,18]), which is unsurprising because organisms are expected to evolve traits that maximize gene propagation at ecologically relevant time scales rather than traits that ensure eternal life. Evolutionary processes driven by natural selection also shed light on the origin of different ageing patterns among organisms [19]. This is because high external mortality makes it less beneficial to allocate resources to anti-ageing mechanisms [9,10,20], including the production of antioxidants [21,22] and chaperones [23–25] that can prevent damage and mechanisms that can repair damage, such as DNA repair enzymes [26–29], autophagy [30,31] and cell/tissue replacement [13,32–34]. As predicted by evolutionary models of resource allocation [8,9,35], natural selection favours organisms that optimize resource allocation to different competing functions, such as growth, reproduction and tissue maintenance [8,13,35–37]. Consequently, ageing patterns should reflect different lifetime schedules of resource allocation, each maximizing fitness under given external mortality conditions (see also [38] on the theory of disposable soma).

**Figure 1.**
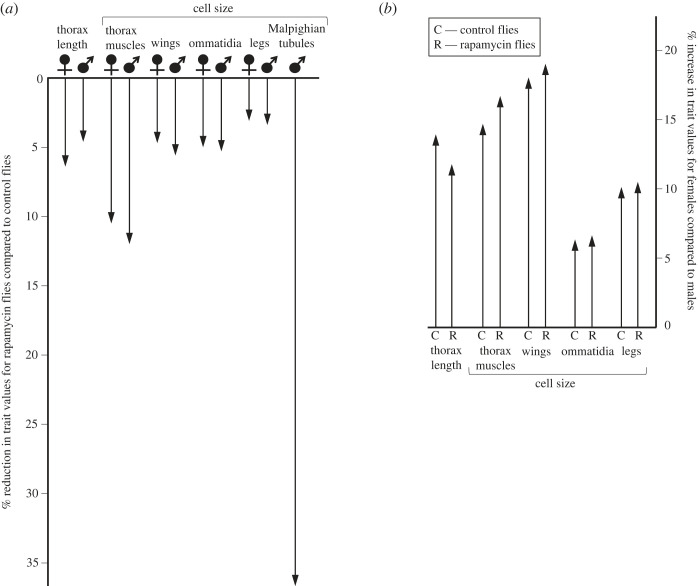
Adult phenotypes of *Drosophila melanogaster* after larval feeding diets with and without rapamycin. (*a*) Female and male rapamycin-treated flies were characterized by a smaller body size and smaller cells in all studied organs compared to control flies. The effect was significant at *p* ≤ 0.0002 for thorax length and the size of cells in muscles, wings, ommatidia and Malpighian tubules but only at *p* = 0.168 for the size of leg cells. (*b*) Independent of diet, females were characterized by a larger body size and larger cells in all studied organs compared with males (*p* < 0.0001). Arrows show mean values obtained from Szlachcic *et al*. [43], estimated with statistical methods from measurements of individuals derived from the same pool of flies as studied here but used to explore orchestration of cell size throughout the body via TOR activity (for detailed statistical results, see table 1 in [43]). Thorax length (mm) was measured as a proxy for body size. Cell size was measured in five (males) or four (females) organs as follows: dorsal longitudinal indirect flight muscle cells in the thorax (µm^2^) by the mean cross-sectional area of fibres; epidermal cells in the wing (µm^2^) by the number of trichomes per unit area; ommatidial cells (µm^2^) by the mean area of ommatidia in the eye; epidermal cells in the leg (µm^2^) by the number of trichomes per unit length; and Malpighian tubule epithelial cells (µm^2^; measured only in males) by the number of nuclei/nucleoli per unit area.

A portion of the molecular work involved in tissue maintenance is allocated to keeping plasma membranes operational, which includes the generation and maintenance of ion gradients on the cell surface and the maintenance of a membrane composition that enables an optimal physical state of membranes [20,39–41]. Interestingly, evidence suggests that up to 5% of genes in a eukaryotic cell may be involved in the synthesis of different types of lipids needed to meet the metabolic requirements of cell membranes [42]. This points to a potential allocative conflict between mechanisms that maintain plasma membranes in the operational state and mechanisms involved in other forms of cellular maintenance, but to the best of our knowledge, this trade-off has not been addressed in previous studies. Addressing this perspective, we studied mortality rates in adult flies of *Drosophila melanogaster* with different cell sizes induced by developmental conditions (figure 1 for phenotypic characteristics of the studied flies). We considered that with a decreasing cell size, the total area of plasma membranes that surround the cytoplasm increases per unit of tissue volume, which should increase costs associated with keeping plasma membranes operational. Indeed, earlier research revealed that species of beetles and birds [44,45] or bacterial populations [46] characterized by smaller cells exhibited higher mass-specific resting metabolic rates. We hypothesized that the increased metabolic demands imposed by keeping more plasma membranes in working order would intensify competition for resources with other cellular activities, such as anti-ageing mechanisms, resulting in higher intrinsic mortality and thus accelerated ageing and shorter lifespans in small-celled flies than in large-celled flies. To obtain adult flies characterized by different cell sizes, we manipulated TOR/insulin signalling pathways involved in cell size control, rearing larvae originating from different genetic lines (isolines) on standard food or on food enriched with rapamycin (figure 1). Rapamycin is a bacterial antibiotic used as an immunosuppressive drug (e.g. [47]). At the molecular level, rapamycin blocks the activity of the target of rapamycin (TOR) protein kinase, inhibiting signal transduction in the TOR/insulin pathways, a backbone of the molecular system of nutrient sensing and cell cycle regulation, autophagy and metabolism [48]. Rapamycin administration to *D. melanogaster* larvae has been shown to lead to delayed development, reduced fly size and, importantly, reduced cell size [43,49–52]. Therefore, we predicted that the rapamycin-treated larvae in our experiment would emerge as adults with smaller cells, which would consequently increase the metabolic cost of these flies in adulthood, leading to increased mortality and thus accelerated ageing and shorter lifespans. Importantly, this effect contrasts sharply with the common image of rapamycin as a promising anti-ageing agent, but we note that these two effects of rapamycin should not be confused. Most of the previous research on rapamycin and ageing considered rapamycin supplementation in later life stages, which has been shown to result in prolonged adult life in flies and mice [53–58]. By contrast, our study appears to be pioneering in this field, as no previous studies have chronically administered rapamycin throughout development and investigated its effects on mortality in adult stages freed from the direct effects of rapamycin.

## 2. Methods

We studied 14 isolines originating from the wild population of *D. melanogaster* at the Jagiellonian University winery (49°58′00.8″ N 20°29′54.1″ E). In September 2017, we transferred gravid wild females to the Institute of Environmental Sciences (Jagiellonian University, Krakow, Poland) to establish a stock of inbred isolines for future studies. All flies were reared in thermal cabinets (POL-EKO Aparatura, Wodzislaw Slaski, Poland) with a 12 h : 12 h L : D photoperiod and stable 70% humidity, which was achieved by placing water containers in the cabinets. For logistical reasons, the cabinets were set to 25°C for isoline establishment and to 20.5°C for the maintenance of stock flies and for creating experimental conditions. The isolines were produced with the help of sib-mated females through 32 generations (for details, see [49]). Flies were reared in 40 ml vials (2.5 cm diameter, 9.5 cm height; polyurethane foam plugs) with 10 ml of cornmeal yeast medium (Bloomington Drosophila Stock Center, Bloomington, IN, USA). Transfers of flies to vials with fresh food were performed every two weeks during isoline establishment and every three weeks under stock conditions to maintain non-overlapping generations.

### 2.1. Induction of adult phenotypes

**Figure 2.**
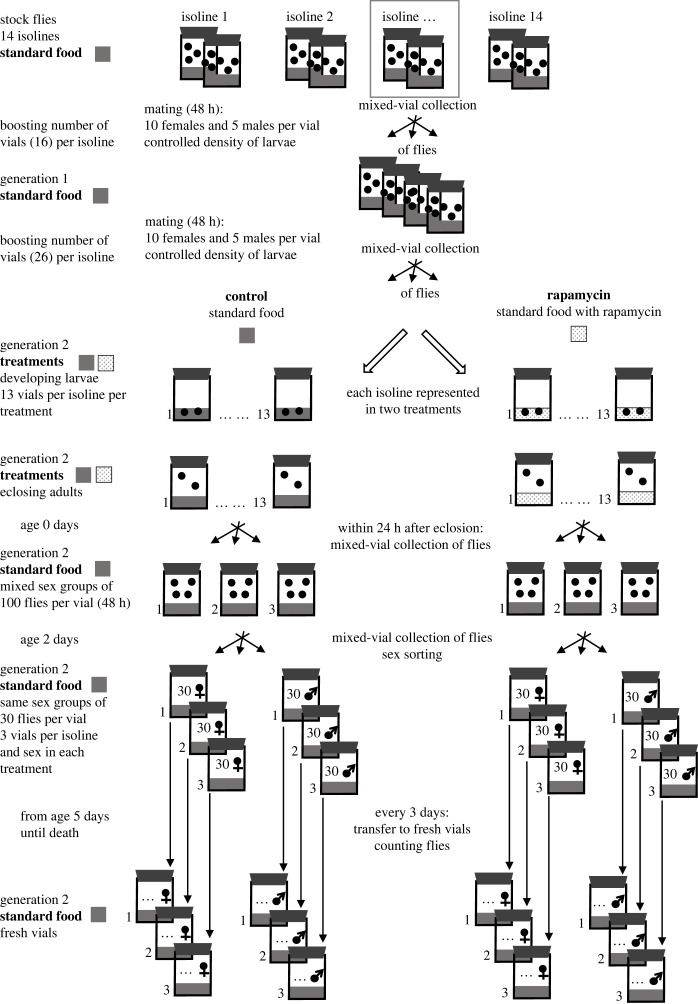
Study design used to raise adult *Drosophila melanogaster* after larval feeding diets with and without rapamycin. Flies from each of 14 isolines were raised on diets with or without rapamycin (rapamycin versus control flies), using procedures shown here for one of the studied isolines. Using stock isolines, we first produced two generations of flies with controlled mating and larval density (generations 1 and 2), increasing the number of vials with flies for each isoline. The second-generation larvae were raised on either standard food or food enriched with rapamycin. Females and males emerging from the two developmental treatments were then maintained in same-sex groups until death on standard food without rapamycin to compare mortality between developmental treatments. We used a mixed-vial collection approach when collecting flies, sampling flies from as many vials as possible for each isoline. Another set of flies originating from the same experiment was used by Szlachcic *et al*. [43] to characterize body size and cell size of the studied flies (figure 1).

Figure 2 summarizes our study design and main procedures. Following our previous methods [49], we produced two cell size phenotypes in adult flies by rearing larvae originating from each isoline on standard food either with or without rapamycin supplementation. The phenotypic induction was preceded by the production of two consecutive generations (two transfers) at controlled density, which boosted the number of flies available for the experiment. When collecting flies, we sampled and mixed flies from as many vials as possible for each isoline, which randomized any potential effects of vial differences on flies (mixed-vial collection). Upon each of the two transfers with density control, groups of 10 females and 5 males from each isoline were placed in large vials (68 ml vials with 20 ml of cornmeal yeast food) for mating and egg laying for 48 h. During the first transfer, 16 vials per isoline were created, and this transfer did not involve experimental treatment (food with and without rapamycin). During the second transfer, the mating groups (again 10 females with 5 males per isoline) were placed into vials with or without rapamycin-supplemented food for egg laying. The second transfer resulted in 13 vials per isoline in each of the two experimental groups. In this way, a new generation of flies was developed on either standard food without rapamycin (control flies) or on standard food supplemented with rapamycin (rapamycin flies). Following [59], rapamycin (Alfa Aesar by Thermo Fisher Scientific, Kandel, Germany) was dissolved in ethanol (Linegal Chemicals, Warszawa, Poland), and the solution was mixed with freshly cooked standard fly medium at a 1 µM concentration. For consistency, the control flies received the same amount of ethanol (but without rapamycin) in their food.

### 2.2. Mortality and longevity

The vials with developing control and rapamycin flies were checked daily for emerging adults (figure 2). Emerging flies were either discarded (if few were available at the time) or collected for the study (if several hundred were available at the time) using an insect exhauster, which ensured collection and mixing of flies from multiple vials per isoline (mixed-vial collection) without anaesthesia. The collected adult flies were transferred to new, large vials (70–100 flies per vial) and fed standard food with no further rapamycin supplementation. The flies were allowed to mate freely for 48 h in groups. Next, the flies were sorted by sex using brief cold anaesthesia and transferred in single-sex groups of 30 ± 3 individuals to six large vials (three vials per sex). All flies remained under our experimental conditions until physiological death. To prevent deterioration of living conditions over time, we transferred the flies every 3 days to new vials with fresh food. Upon each transfer, we counted dead individuals as well as all escaped or accidentally killed flies, which were then used as censored data in our statistical analysis. To minimize the risk that flies would become stuck in the food, the vials with flies were maintained in a horizontal position throughout the experiment. At the end of the experiment, we pooled data from all three vials representing each isoline, sex and diet treatment, starting with approximately 90 flies per isoline, sex and treatment, to compare mortality and survivorship between treatments.

### 2.3. Statistical methods

All analyses were performed using R (v4.0.2) software [60]. We began our analysis with a standard approach by calculating and plotting Kaplan–Meier (KM) estimators of survivorship to visualize the effects of treatment, sex and isoline on survival (*survival* v3.2–13 and *rms* v6.2–0 packages [61,62]). Then, to compare survivorship curves, we performed a non-parametric log-rank test. We used the stratified Gehan–Breslow test (Hothorn–Lausen ties method, *coin* package [63]) for female versus male comparisons and for control versus rapamycin treatment comparisons. Stratification was used to control for the effects of other variables: treatment or sex (depending on the comparison). The Gehan–Breslow test is more sensitive to initial differences in survival distributions, which were visually observed in our KM plots, and it is more tolerant to deviations from a proportional hazard assumption (our data violated this assumption) and to survival curves intersecting near the median lifespan (which occurred between some of our curves). Survivorship is a cumulative measure, as by definition, it integrates the effects of all changes in mortality until a specific age. For example, an increase in mortality at earlier ages can still be observed in the survivorship curve at late ages. From the biological perspective, it is often more interesting to investigate non-cumulative age-specific effects, which are easier to interpret using mortality analysis. For this purpose, we used parametric generalized additive mixed models (GAMMs, *mgcv* v1.8–36 package [64]), which analyse differences in (log) mortality rates. GAMMs are an extension of generalized linear mixed models (GLMMs) and use penalized smoothing functions for some predictor variables (smooths) to model nonlinear patterns. The mortality rate in generalized models is modelled using count data, with the number of deaths as the dependent variable and the offset set to the log of days lived for individuals (exposures, classic life table approach). We considered four fixed terms across all tested models: sex, treatment, their interaction and the smoothing functions of age modelled separately for each combination of sex and treatment. The random effects of isolines were modelled via factor smooth interactions, and model selection was performed using Akaike information criterion (AIC) methods (e.g. [65]).

The most parsimonious GAMM was used to calculate marginal mortality rates (marginal hazards). Marginal mortality is based on the concept of heterogeneity in frailty [66]. It is expected that individuals (or genotypes) with the highest frailties will die earlier, changing the composition of the population over time and thus affecting the marginal mortality rate. In our work, the concept of the marginal mortality rate (and the corresponding marginal survivorship) is used to compare mortality rates of different combinations of sex and treatment that include the random effects of isolines as well as to test the goodness of fit. To control our analyses for the effects of heterogeneity in the frailties of isolines, we removed random effects associated with isolines from model predictions but not from the model itself. This allowed us to determine the ‘baseline’ mortality rates (predictions conditional on random effects) of different combinations of sex and treatment as well as their differences. The piecewise confidence intervals of the differences were calculated using the bootstrap percentile method (e.g. [67]). More details on the GAMMs can be found in the electronic supplementary material.

## 3. Results

**Figure 3.**
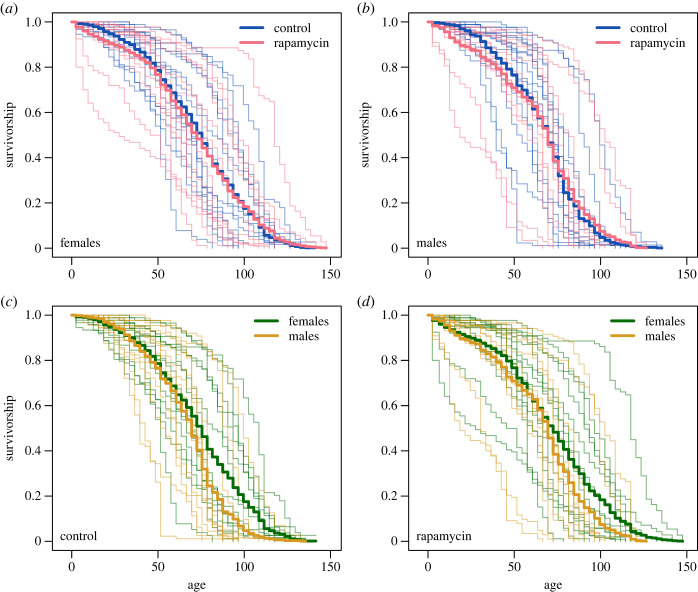
Survivorship curves of adult *Drosophila melanogaster* flies estimated by the KM method. Thin lines indicate survivorship of each isoline, and thick lines indicate survivorship of different combinations of sex and treatment. Flies were raised by larval feeding on diets with or without rapamycin (rapamycin versus control flies), but in adulthood, all flies were fed standard food. Initially, each isoline was represented in each treatment by approximately 90 adult males and 90 adult females. The phenotypic characteristics of flies are shown in figure 1. Panels show survivorship of (*a*) control and rapamycin females; (*b*) control and rapamycin males; (*c*) females and males in the control; (*d*) females and males in the rapamycin treatment. Small cell size phenotypes (rapamycin-treated flies) are associated with lower survivorship at a young age than large cell size phenotypes (control flies). Females have higher survivorship than males.

**Figure 4.**
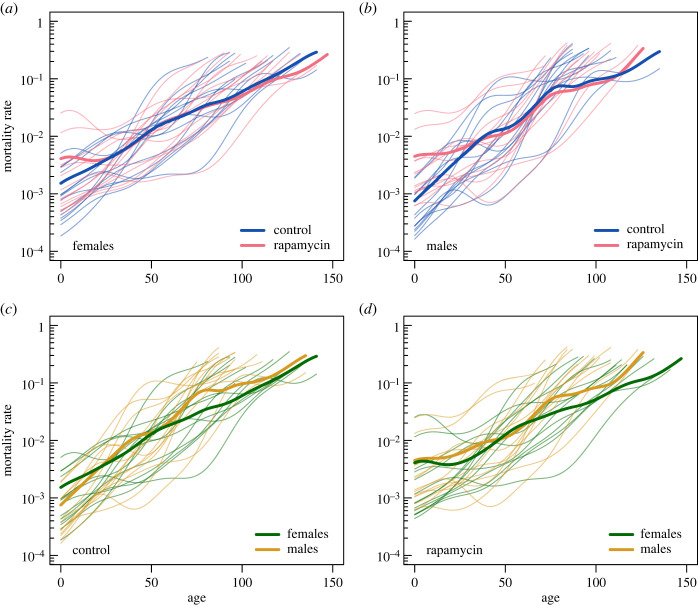
Mortality rates of adult *Drosophila melanogaster* flies estimated by GAMMs. Thin lines indicate the predicted mortality rates of each isoline, and thick lines indicate the marginal predicted mortality rates. Flies were raised by larval feeding on diets with or without rapamycin (rapamycin versus control flies), but in adulthood, all flies were fed standard food. Initially, each isoline was represented in each treatment by approximately 90 adult males and 90 adult females. The phenotypic characteristics of flies are shown in figure 1. Panels show the mortality rate of (*a*) control and rapamycin females; (*b*) control and rapamycin males; (*c*) females and males in the control; (*d*) females and males in the rapamycin treatment. All predictions are plotted within observable age ranges. The mortality rate associated with the small cell size phenotype (rapamycin-treated flies) was higher at the beginning of adult life than that of the large cell size phenotype (control flies). Males have a higher mortality rate than females.

**Figure 5.**
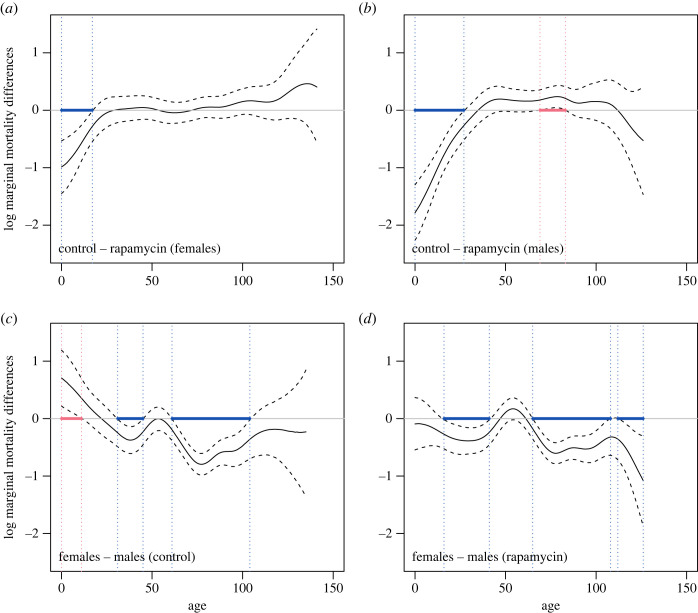
Differences in predicted log marginal mortality rates of adult *Drosophila melanogaster* flies calculated from GAMM estimates. Differences in log marginal mortality rates (which are affected by compositional changes of isolines due to heterogeneity in frailty among isolines; see supplementary material for details) between (*a*) control and rapamycin females; (*b*) control and rapamycin males; (*c*) females and males in the control; (*d*) females and males in the rapamycin treatment. The solid line shows differences; dashed lines show 95% confidence intervals; blue and red lines show age ranges for which the differences become significant (at the level of 0.05), negative and positive, respectively. All log marginal differences in mortality rates are plotted within observable age ranges. Flies were raised by larval feeding on diets with or without rapamycin (rapamycin versus control flies), but in adulthood, all flies were fed standard food. Initially, each isoline was represented in each treatment by approximately 90 adult males and 90 adult females. The phenotypic characteristics of flies are shown in figure 1.

KM estimators of survival (figure 3) and stratified Gehan–Breslow tests (electronic supplementary material, table S1) showed that flies reared on food with rapamycin had lower survivorship at young adult ages than control flies (figure 3*a*,*b*; electronic supplementary material, table S1, *p* = 0.0458), with males having lower survivorship than females (figure 3*c*,*d*; electronic supplementary material, table S1, *p* < 0.0001). The initial survivorship differences between rapamycin-treated and control flies disappeared later in life, as the survivorship curves in figure 3 overlap in older flies. Indeed, when we compared the marginal mortality curves (figure 4*a*,*b*) obtained from GAMMs, we found that while the mortality curve for rapamycin was shifted upwards at the beginning of life, it dropped down and overlapped with the control curve later in life. The lower survivorship of males than females was maintained throughout almost the whole lifespan (figure 3*c*,*d*) and resulted from lower marginal mortality rates in females than in males (figure 4*c*,*d*). Figure 3 also shows large variation in survivorship among isolines. Consistently, our GAMM showed inter-isoline variation in mortality curves (figure 4; electronic supplementary material, figure S2e–h). All investigated groups (treatments and sexes) showed clear ageing because marginal mortality rates increased roughly exponentially with age, as indicated in figure 4 by semi-linear trends in log mortality values. Interestingly, the similar slopes of the marginal log mortality trends indicated similar acceleration of ageing in all studied groups. However, mortality patterns clearly differed between treatments and sexes, as the interaction between treatment, sex and age was an important component of the model (electronic supplementary material, tables S2 and S3). Mortality data shown in figure 4 were further analysed statistically using between-group differences in log marginal mortality rates (figure 5). At the beginning of life (up to 17 days), rapamycin females had higher log marginal mortality rates than control females (figure 5*a*). A similar pattern was observed for males for a longer time (up to 27 days), but this pattern was reversed at 69–83 days, such that rapamycin males had a lower log marginal mortality rate than control males (figure 5*b*). Although females from the control treatment had a slightly higher log marginal mortality rate at the beginning of adult life (up to 11 days) than control males, they had a much lower mortality rate for most of their life (significant differences at 31–45 and 61–104 days of life; figure 5*c*). Similar sex differences were observed for the rapamycin treatment, where females had lower log marginal mortality than males for most of their life (significant differences at 16–41, 65–107 and 113–126 days of life; figure 5*d*).

**Figure 6.**
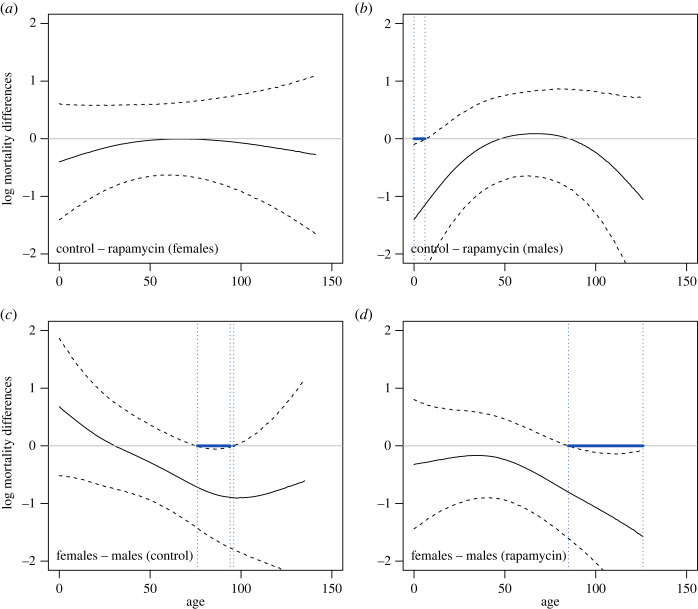
Differences in predicted log mortality rates of adult *Drosophila melanogaster* flies calculated from GAMM estimates. Differences in conditional log mortality rates (after excluding effects of heterogeneity in frailty of isolines from model predictions) between (*a*) control and rapamycin females; (*b*) control and rapamycin males; (*c*) females and males in the control; (*d*) females and males in the rapamycin treatment. Solid lines show the predicted differences; dashed lines show 95% confidence intervals; blue lines show age ranges for which the differences become significant (at the level of 0.05). Flies were raised by larval feeding on diets with or without rapamycin (rapamycin versus control flies), but in adulthood, all flies were fed standard food. Initially, each isoline was represented in each treatment by approximately 90 adult males and 90 adult females. The phenotypic characteristics of flies are shown in figure 1.

Importantly, the patterns in marginal mortality shown in figures 4 and 5 could inherently be driven by compositional changes in isolines in the studied groups. Therefore, in the next step, we ‘controlled’ for this effect by performing mortality analysis again using the so-called conditional log mortality rates (figure 6). In other words, we investigated log mortality differences between the rapamycin and control treatments, limiting the effect of population processes driven by heterogeneity in frailties. The log mortality differences between the control and rapamycin groups presented a hump shape (figure 6*a*,*b*), with peak values either approaching zero (females) or slightly exceeding this value (males). Despite the fact that our 95% confidence intervals were wide and included zero (for mortality differences), this pattern indicates that throughout almost the entire lifespan, mortality rates tended to remain higher in rapamycin-treated flies than in control flies (negative values of the differences), but these differences disappeared (females) or even reversed (males) for a relatively brief period of time in the middle of adult life. While this analysis could not statistically detect differences in early log mortality rates of females between the control and rapamycin treatments (figure 6*a*), we found such differences in very early adulthood in males (figure 6*b*). Figure 6*c*,*d* shows that, in general, differences in log mortality rates between females and males decreased with age, indicating that females died more slowly than males. Notably, for each treatment, a significant difference in log mortality rates between males and females occurred at different ages, i.e. 76–96 days for control flies (figure 6*c*) and 85–126 for rapamycin-treated flies (figure 6*d*).

## 4. Discussion

In all groups of *D. melanogaster*, the mortality rate accelerated approximately exponentially with age, showing a Gompertz-type ageing pattern [68]. Among the different ageing patterns observed in nature [19], the Gompertz-type pattern has been described, for example, in humans [68–70], non-human primates [71], rodents and birds [72], and some invertebrates such as *Daphnia* [73,74] and *Drosophila* flies [75,76]. Our data revealed differences in mortality rates and survivorship among the isolines of flies. Such heterogeneity is thought to affect estimates of marginal mortality rates, leading to irregularities in mortality and survival patterns, such as a sudden deceleration of mortality rates early or late in life (e.g. [77–79]), and these irregularities also occurred in our data (figures 3 and 4). Interestingly, the mortality differences among isolines that originated from a wild population in our study suggest a high level of genetic variance in this trait. This finding supports previous views that despite a strong association with Darwinian fitness, many morphological, behavioural and life-history traits retain high levels of genetic variance in wild-type *Drosophila* populations [80–82]. Nevertheless, previous studies revealed that *Drosophila* life-history traits often show low heritability [36,83,84], which indicates a relatively large contribution of environmentally induced trait variance to phenotypic differences between individual flies [82]. We also note that our study involved only 14 different genotypes, which seems to be too few to obtain conclusive results on the level of genetic variance in the studied traits.

Our experimental results showed that feeding larvae food enriched with rapamycin resulted in adult flies with worse survival than flies without prior exposure to rapamycin. This suggests that prolonged administration of rapamycin throughout development may increase intrinsic mortality rates in adulthood. Such an effect of rapamycin has never been demonstrated, but it should be emphasized here that previous studies did not measure the consequences of rapamycin administered throughout development on the survival and physiology of adult forms following rapamycin withdrawal. Most previous research in this field has focused on the direct effects of rapamycin administered to adult flies, demonstrating reduced adult mortality as a result of this treatment (e.g. [53,55,58]). Interestingly, in contrast to our results, it has been recently shown that transient exposure of the last larval stage (stage 3) of *D. melanogaster* to a rapamycin diet improves the survival of eclosing adults, but this effect was imposed only by the highest dose of rapamycin (200 µM), while lower doses of 50 µM or 1 µM (we used a 1 µM dose) showed no effect on adult survival [85]. We note, however, that larvae in our experiment were continuously exposed to rapamycin from the egg stage to the pupa and adult emergence stages, so the two experiments are not fully comparable. Obviously, we cannot rule out that, following the known direct effects of rapamycin, rapamycin administration during development in our study prevented mortality in some handicapped larvae, allowing them to metamorphose. As a result, compared to our control group, eclosing flies in the rapamycin-treated group could have been enriched in high-frailty flies, which died when the rapamycin ‘mortality umbrella’ was removed. Altogether, looking at our data and previous rapamycin-oriented studies, we conclude that the decreased viability of rapamycin-treated flies in our experiment cannot be simply deduced from extrapolating the well-known direct effects of rapamycin on physiology. Here, instead of invoking the direct metabolic effects of rapamycin, we consider its potential indirect effects mediated by rapamycin-induced changes in the cellular composition of the body. Rapamycin downregulates the activity of TOR pathways, leading to reduced cell size in tissues [49,52]. Indeed, our recent analysis of cell size measurements collected from the siblings of the flies studied here showed a highly coordinated reduction in cell size in different tissue types and organs of adult flies in response to larval feeding on rapamycin [43], ranging from a size reduction of 3.1% in epidermal cells in the legs to as much as 36.7% in epithelial cells in Malpighian tubules (figure 1*a*). Interestingly, such cellular effects of rapamycin occurred in a similar manner between the organs of both males and females (figure 1*b*). Taken together, the available evidence is very suggestive that the metabolic effects of smaller cells should be considered in explaining the reduced viability of adult flies originating from our rapamycin treatment in the experiment. We suggest that the metabolic demands of maintaining plasma membranes may compete for resources with other maintenance functions, leading to decreased viability in small-celled organisms that maintain more plasma membranes in tissues than large-celled organisms. Following the theory of optimal cell size (TOCS), the cellular composition of an organism, i.e. the size and number of cells in tissues, is not a neutral trait, as it is associated with costs and benefits [20,39,40,44,86–96]. On the one hand, the small volume of cytoplasm handled by nuclei, short within-cell distances and large surface area of plasma membranes make small cells advantageous in terms of their transport capabilities. For example, Verspagen *et al*. [97] showed that flies with smaller cells survived longer under acute, intense heat stress than flies with larger cells. On the other hand, costly maintenance of plasma membranes would divert resources from other functions, which in light of our work, may, for example, lead to accelerated intrinsic mortality. Importantly, supporting the TOCS, the costs and benefits of cell size balance out differently depending on metabolic demand and supply. For example, Szlachcic & Czarnoleski [49] showed that small-celled flies resulting from larval food supplementation with rapamycin were less oxygen-limited during flight than large-celled flies when body temperature approached a physiological thermal optimum, but this advantage disappeared at temperatures approaching physiological thermal limits. Walczyńska *et al*. [88] showed that small-celled freshwater rotifers had higher reproductive performance than large-celled rotifers in warm hypoxic waters but not in warm normoxic, cool normoxic or cool hypoxic waters. Complementary to the mechanisms inferred here from the TOCS, peroxidation of plasma membrane phospholipids by oxygen free radicals may be involved in the association between cell size and intrinsic mortality. The accumulation of molecular damage associated with phospholipid peroxidation is thought to be involved in ageing, and in line with this, species or genetic forms characterized by longer lifespans often show less of this type of damage [98,99]. We therefore predict that a reduction in cell size, which increases the area of plasma membranes in the body, will increase the total amount of phospholipids exposed to free radical damage, which in turn may contribute to the sources of intrinsic mortality directly or indirectly by increasing the need for damage repair and consequently depleting cellular resources via mechanisms involved in plasma membrane maintenance.

Our results agree with our original hypothesis that small-celled flies are at increased risk of death but do not support our predictions that these effects are maintained throughout life, which would lead to accelerated ageing and shorter lifespans. Notably, the effects of impaired survivorship of small-celled flies (our rapamycin treatment) persisted over a short initial period in the adult life of flies, at best for 30–50 days after eclosion, and disappeared in older flies. The mechanism responsible for this change needs to be further investigated, although we can speculate on two potential scenarios. One likely possibility is allocative trade-offs with other organismal functions that have not been explored here. For example, at the beginning of adult life, flies maintain high physiological activity of reproductive functions, so at this stage, it may be particularly difficult to reconcile expenditure on other functions, including those related to the maintenance of plasma membranes. Another rationale invokes population processes, proposing a mechanism non-mutually exclusive to other explanations. It is important to consider that, in nature, a number of genetic and developmental factors result in adult flies having unequal 'frailty', with flies of the highest frailty dying earlier than flies of lower frailty. As a result, populations are depleted of flies with the highest frailty over time. Thus, if our control and rapamycin groups were mixtures of flies with different frailties, and additionally our rapamycin flies were eclosing with smaller cells that increased metabolic living costs, then deaths of high-frailty flies were likely shifted towards early adult life in our rapamycin treatment compared to the control. Once the abundance of the frailest individuals diminished in the rapamycin group, both the control and rapamycin groups manifested less pronounced differences in mortality rates. Because this effect occurred early in life and the survivorship is a cumulative function of mortality, the curves for the rapamycin and control groups became similar later in adult life (see [79] for a theoretical discussion of the effect of heterogeneity in frailty on mortality and survivorship patterns). Putting aside the discussion of the factors that shaped the mortality pattern in our experiment, it is worth noting here that the time window in which we observed the strongest differences in survival between rapamycin-treated and control flies (the first couple of days after eclosion) appears to be highly ecologically relevant, as the expected lifespan of wild adult flies is approximately 11–14 days [100]. Therefore, the cell size effects on mortality suggested by our study may correspond to the efficiency of gene transfer to the next generations in natural populations of *Drosophila* and thus be under the control of natural selection. Obviously, sources of intrinsic mortality that impose measurable effects under the stable and benign conditions of laboratory experiments are likely to exacerbate their effects under natural ecological conditions. For example, compared to the small-celled flies in the laboratory experiment, their wild counterparts would have to cope with the metabolic demands of locomotion (flight) and remodelling of membrane composition to maintain proper fluidity in the face of thermal fluctuations.

It is worth looking further at the discrepancy between our results and those of previous studies showing improved adult survival resulting from rapamycin administration [53,55] and dietary restriction [101,102] in adulthood or genetic mutations in the TOR/insulin signalling pathways [103–105]. These contrasting data, although they may initially appear puzzling, are consistent with our current understanding of the links between the activity of nutrient sensing pathways and the life cycles of organisms. Stimulation of the TOR/insulin pathways by signals about nutrients reduces autophagy and increases cell proliferation, which mechanistically leads to an increase in cell mass that supports juvenile growth and development [106–108]. However, persistent activation of the TOR/insulin pathways in adulthood is thought to be one of the main drivers of ageing [14]. This explains why slower ageing may be the result of the silencing of the TOR/insulin pathways in the adult body by mutations that act throughout life [103–105] or by the use of rapamycin or a low-calorie diet in adulthood [53,55,101,102]. By contrast, in our study, we did not focus on the direct effects of altered activity of nutrient-sensing pathways but on the consequences of adult phenotypes (here, reduced cell size and perhaps other unmeasured characteristics) that were triggered by transient changes in the activity of these pathways during development. The contrasting direct and indirect effects of the activity of nutrient sensing pathways on intrinsic mortality may also help better understand the inconsistencies in the relationship between body size and lifespan in nature. Between species, as documented, for example, in birds and mammals, ageing is often faster in smaller species [109], and emerging evidence suggests that small mammalian and avian species are characterized by smaller cells in tissues, which are accompanied by faster mass-specific resting metabolic rates [44]. This pattern is consistent with the effect of cell size and plasma membranes on metabolic costs and ageing postulated in our work, but it is striking that this resemblance becomes even stronger when we also consider body size differences between the studied flies. Notably, our rapamycin-treated flies were characterized not only by reduced cell size and increased mortality in early adulthood but also by a 5–6% smaller adult body size compared to that of control flies (figure 1). In turn, many within-species data show that smaller individuals, as documented, for example, in dogs [110] and mice [111,112], often live longer than larger individuals, leading to an inverse relationship between body size and longevity. From an evolutionary perspective, a positive relationship between body size and longevity on an inter-species scale should emerge from the promotion of late maturation and large and long-lived adults in safe environments and early maturation and small and short-lived adults in unsafe environments [8] (see also [113] for a wider discussion). Following Blagosklonny [114], if larger species are selected for increased longevity, they should show reduced activity of nutrient-responsive pathways when they reach maturity. Indeed, compared to those of large and long-lived species, the pancreatic cells of small and short-lived species had enlarged cytoplasm and nucleoli, indicating increased endocrine activity in this organ and thus upregulation of TOR/insulin pathways throughout the body [115]. By contrast, at the intraspecific scale, fast-growing organisms would represent forms with permanently hyperactive nutrient sensing pathways, maturing early with large body size at the expense of accelerated ageing later in life. Indeed, mutations in the CHICO insulin receptor protein cause increased lifespan and reduced adult body size in *Drosophila* flies [103,116]. Interestingly, *chico* mutants are also characterized by reduced cell size in the body [117], but we note that this does not appear to contradict our predictions for the effect of cell size on longevity. This is because mutations in nutrient-sensing pathways act over the entire lifespan, exerting direct effects (e.g. inhibiting autophagy) in adulthood.

Our study included males and females, which allowed us to consider sex-specific mortality patterns in *D. melanogaster*. We found no initial sex-related differences in mortality, such that males and females reached comparable median life expectancies, but after this stage, males apparently experienced higher mortality than females, which resulted in fewer males surviving to old age. Similarly, Lints *et al*. [118] reported that female *D. melanogaster* flies can be characterized by longer maximal but not necessarily mean lifespans than male flies. Our results are consistent with the general pattern described for vertebrates and invertebrates that the homogametic sex (females in the case of *Drosophila*) tends to live longer than the heterogametic sex [119]. Interestingly, previous studies have shown that male *D. melanogaster* have smaller cells in the body than females [93,120]. Indeed, following the results of Szlachcic *et al*. [43], females eclosed in our experiment with 12–14% larger body sizes and 6–19% larger cells in different organs compared to those of males (figure 1*b*). Therefore, it is worth noting that sex differences in survival and cellular phenotype in *D. melanogaster* are consistent with the links proposed here between cell size and ageing, although we emphasize that there is a wide range of factors related to sex that probably shape sex differences in survival in the first place. Interestingly, comparisons of different species of carabid beetles showed that females and males did not systematically differ in cell size and resting metabolic rate [45]. However, the sex with smaller cells, regardless of whether in a given species it was the female or male, had higher resting metabolic rates than the sex with larger cells. These findings suggest that the differences between sexes in living costs can be related to the cellular composition of the body of each sex but not necessarily to sex alone. This suggests some new explorative avenues in research aimed at the identification of different drivers of sex differences in living costs and intrinsic mortalities. If the results of our study were applied to ecologically relevant *Drosophila* lifespans, the detected sex differences in survival would appear to have no significant effect on evolutionary processes in natural populations. However, we note that sex differences in *Drosophila* survival can be shaped by a number of different factors, including mating intensity [121–123], and our study was not designed to account for such effects. Sex differences in survival show complex patterns that are strongly condition-dependent [121,124], which might help explain some inconsistencies in published evidence on sex differences in flies and other ectotherms [118,121].

## 5. Conclusion

Overall, our results are important for better understanding mortality patterns in nature. Here, we present evidence suggesting links between cell size and intrinsic mortality, concluding that differences in cell size between organisms may be an important but overlooked factor affecting the risk of mortality. These results are highly suggestive, but at this point, we are far from understanding the precise molecular mechanisms that would determine the mortality consequences of cell size variation. Here, we invoke the importance of an allocative trade-off between functions preventing intrinsic mortality and mechanisms involved in the maintenance of plasma membranes, which should change with the cellular composition of the body. Importantly, although we found support in the data for the hypothesis that the cellular composition of an organism can influence its mortality rate, we did not find conclusive evidence that these effects are consistently maintained throughout life, leading to accelerated ageing and shorter lifespans. However, it would be premature to completely abandon this scenario, especially in the face of available but still very scarce evidence on inter-species and inter-sex patterns in cell size, metabolic rates and ageing. Future studies should use different study systems and approaches to test in a rigorous way potential links between the cellular composition of organisms and mortality patterns. Even without conclusive evidence for the effects of cell size on ageing, our results are important for research programmes aimed at the pharmacological prevention of ageing in humans. Previous research on ageing has mainly focused on humans and some short-lived laboratory models, such as flies [125]. Ageing is the main cause of age-related diseases in humans, which is why most contemporary research on ageing focuses on improving human health [2]. Rapamycin, which was used in our experimental study to induce changes in cell size, is considered a promising anti-ageing drug [2] and is already used in human pharmacotherapy for the treatment of many diseases and for the prevention of organ transplant rejection [47]. Despite abundant evidence showing that rapamycin intake leads to extended health and lifespan in various species, it is still not used as an anti-ageing drug in humans due to concerns about its potential negative side effects [126]. According to Potter *et al*. [50], there is a lack of information on the deferred effects of rapamycin administered to developing organisms, making it difficult to determine whether rapamycin or its analogues can be safely used in children and adolescents. Our study appears to be one of very few (e.g. [49,50]) that have addressed such effects, and our results suggest using rapamycin with caution, showing that rapamycin administration to juveniles could increase the risk of death in adulthood. However, it remains to be discovered which physiological mediators are involved in such adverse effects of rapamycin beyond the increased metabolic costs associated with the rapamycin-induced changes in cell size postulated in our study.

## Data Availability

The data are provided in electronic supplementary material [127].
